# Toxicity and Bioaccumulation of Heavy Metals in Spinach (*Spinacia oleracea*) Grown in a Controlled Environment

**DOI:** 10.3390/ijerph120707400

**Published:** 2015-06-30

**Authors:** Naz Alia, Khan Sardar, Muhammad Said, Khalid Salma, Alam Sadia, Siddique Sadaf, Ahmed Toqeer, Scholz Miklas

**Affiliations:** 1 Department of Environmental Sciences, University of Haripur, Haripur 21120, Pakistan; E-Mail: aliaawkum@gmail.com; 2 Department of Environmental Sciences, University of Peshawar, Peshawar 25120, Pakistan; E-Mail: sardar.khan2008@yahoo.com; 3 Department of Earth Sciences, COMSATS Institution of Information Technology, Abbottabad 22060, Pakistan; E-Mail: saidmuhammad1@gmail.com; 4 Prime Institute of Public Health, Peshawar 25120, Pakistan; E-Mail: anagalious_79@yahoo.com; 5 Department of Microbiology, University of Haripur, Haripur 21120, Pakistan; E-Mail: sadia.alam2004@gmail.com; 6 Department of Forestry and Wildlife Management, University of Haripur, Haripur 21120, KPK, Pakistan; E-Mail: dua632@yahoo.com; 7 Centre for Climate Research and Development (CCRD), COMSATS Institute of Information Technology (CIIT), Chak Shahzad, Islamabad 45550, Pakistan; E-Mail: toqeer.ahmed@comsats.edu.pk; 8 Civil Engineering Research Group, School of Computing, Science and Engineering, The University of Salford, Salford M5 4WT, UK

**Keywords:** bioaccumulation, cadmium, contamination, irrigation, lead, nutrient, spinach, toxicity, water resources management, zinc

## Abstract

The impact of heavy metal toxicity on the shoot and root lengths, total protein, fiber characteristics, moisture content and nutrient composition of spinach (*Spinacia oleracea*) was evaluated. Plants were grown in pots containing soil and treated with different concentrations (mg/kg) of lead (Pb; 300, 400 and 500), cadmium (Cd; 0.5, 1 and 1.5) and zinc (Zn; 250, 500, and 700) as well as mixtures of Cd and Pb (0.5/300, 1/400, 1.5/500), Cd and Zn (0.5/250, 1/500, 1.5/700), and Pb and Zn (300/250, 400/500, 500/700). Soil contaminated by long-term irrigation with wastewater containing heavy metals was simulated. An increase in concentrations of heavy metals both individually and as mixtures significantly (*p* < 0.05) reduced the growth parameters and nutrient contents of *S. oleracea.* The uptake patterns of heavy metals in mixtures showed antagonistic impacts on each other. The toxicities of the mixtures Cd and Pb, Cd and Zn as well as Pb and Zn were higher than those observed in separate heavy metal applications but less than their additive sums. The toxicity caused by individual heavy metals was the highest for Cd followed by Pb and Zn. The highest toxicity was observed in plants grown in soil contaminated by Cd and Pb.

## 1. Introduction

Heavy metal accumulation in soil interrupts the normal functioning of soil ecosystems and plant growth [1,2]. Plants absorb various kinds of heavy metals when available in the soil or irrigation water [3]. Metals like manganese (Mn), magnesium (Mg), copper (Cu) and iron (Fe) is classified as plant essential metals. These metals are required in specific amount and their deficiency or elevated concentrations will result in toxic effects and reduce the plant productivity. For example, Mn is involved in splitting water molecules necessary for photosynthesis. Other metals like magnesium deficiency is responsible for cholorosis in plant leaves [4,5] and also induces oxidative stress [6]. Zinc (Zn) is essentially required for plants. However, too high concentrations can damage plants [4] and inhibit their growth. Zinc is responsible for chlorosis in leaves by reducing chlorophyll [7]. However, heavy metals including Cd and Pb are toxic metal and influence the plant growth adversely by affecting the leaves and root growth and inhibit enzymatic activities and resulted in reduce production [8,9].

Cadmium is considered as phytotoxic as it inhibits plant growth parameters including respiration, photosynthesis and water and nutrient uptake [10]. Further it reduces the rate of new cell production and root growth [11], inhibits the ant oxidative enzymes activities [12] and induces oxidative stress in cells [13]. Moreover, Cd induces changes in plants at all biochemical, physical and genetic levels, which are responsible for the reduction in the growth of plants [14], leaf chlorosis, and leaf or root necrosis [15] and ultimately plant death occurred [16]. Like Cd, Pb is also phytotoxic in nature. It affects the plants photosynthesis by reducing the chlorophyll content. This is because Pb reduces the uptake of chlorophyll-essential elements such as Mg and Fe, affecting chloroplast, changing essential enzymatic processes for photosynthesis and disturbing the closing of stomata [17]. Lead has significant impacts on seedling dry mass, root and shoot length, and weight [18,19]. It adversely affects the process of respiration and metabolism of plants [20].

Soils are contaminated in the environment with a number of heavy metals by natural (weathering and erosion of parent rock material or ore deposits) or artificial (wastewater irrigation, mining activities) sources. The presence of one contaminant can increase or decrease the impacts of others. To date, majority of studies have focused or investigated the effects of a single metal on plant species [21,22,23]. However, the study of plant to a mixture of heavy metals requires more attention throughout the world*.*

Human exposure via the oral pathway (*i.e.*, eating food) is one the major routes for heavy metal exposure [24]. *Spinacia oleracea* is a member of the Caryophyllales order, comprising broad, green and leafy vegetables possessing large surface areas, relatively high growth rates and rather elevated heavy metal absorption rates. Recently, due to these unique characteristics, *S. oleracea* and other members of the Caryophyllales order have been researched in a number of scientific studies to observe their growth and toxicity responses to heavy metal contaminations [25,26,27,28]. *Spinacia oleracea* has an imperative position in the order due to large and expanded leafs, fast growth and by being a common part of the human diet. Nevertheless, there is a lack of information regarding growth behavior, metal accumulation, total protein content, fiber characteristics, moisture content and inorganic nutrients response to individual and combined heavy metals with respect to this plant. Therefore, it is necessary to unravel the response of *S. oleracea* to a range of individual and combined heavy metals.

## 2. Materials and Methods

### 2.1. Experimental Design

*Spinacia oleracea* was taken as a representative plant for broad leafy vegetables. Soil contaminated by long-term irrigation with wastewater containing heavy metals was simulated. This is a common practice considering that a field experiment with contaminated irrigation water would otherwise take years or even decades to complete [26]. Soil used was characterized by the following parameters: pH (6.7), organic matter content (2%) and electric conductivity (2.1 dS/m). The soil particle size distribution was as follows: <20 µm (8.15%), ≥20–62 µm (47.3%), ≥62–250 µm (44.0%) and ≥250 µm–1mm (0.34%).

**Figure 1:**
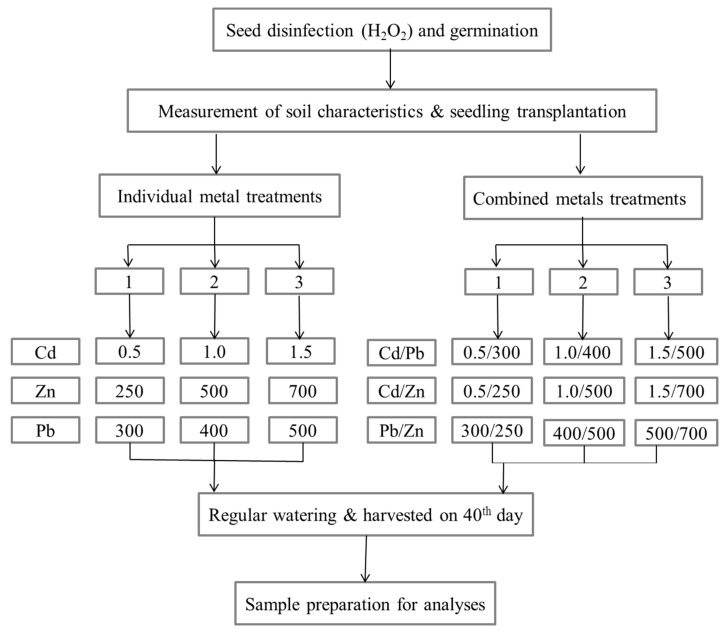
Diagrammatic representation of experimental design and procedure (Cd, cadmium; Pb, lead; Zn, zinc).

Figure 1 shows a diagrammatic representation of the experimental design and key procedures undertaken. Soil from an uncontaminated site was analyzed for physicochemical properties and selected heavy metals to establish a baseline. Representative soil samples were freshly spiked with salt solutions of heavy metals (Cd, Pb and Zn) and thoroughly mixed (all concentrations in mg/kg) according to Figure 1.

Plastic pots were filled with 1 kg of freshly spiked soil having four replicates of each treatment and a control, and marked properly. The disinfected (30% (w/w) hydrogen peroxide solution for 10 min) seeds were germinated in Petri dishes inside the folds of wet filter papers at 28 ± 1 °C. After four days, five uniform seedlings were cultivated in each plastic pot. This experiment was performed in a greenhouse with a day temperature of 25 ± 4 °C and a night temperature of 19 ± 3 °C. The plants were kept under sunlight for 12 h and at a relative humidity of 65% ± 2%. The positions of pots were changed on a regular basis to ensure similar light and temperature readings. The plants were irrigated with deionized water (100 mL) twice per day and were harvested after maturation at 40 days. The plants were washed with tap water and then deionized water and were cut down into shoots and roots. Fresh weights as well as length and shoot diameters and other physical appearance were recorded. Roots and shoots were oven dried at 70 ºC for 48 h and powdered with grinder and sieved through 2 mm mesh size [24,29].

Moisture, fiber and protein contents were determined using standard methods [30]. Moisture was analyzed by using the dry oven method. A plant sample was dried in an oven at 105 °C for 3 h. The Kjeldahl method was used for evaluating nitrogen and subsequently the protein content by calculation. The crude fiber content was estimated according to Aldwairji *et al.* [31], who developed a method based on a procedure by the Association of Official Analytical Chemists [32]. The total protein and fiber content of each treatment (including the control group) were determined. Reductions of protein and fiber contents subject to treatments were compared with the control group to assess the impact of the corresponding treatment.

### 2.2. Metal Extraction and Analysis

Plants were weighed and samples of 0.5 g were put in acid-washed and dried digestion tubes. Powdered plant parts were mixed with (15 mL) of nitric, perchloric and sulfuric acids (5:1:10) overnight to prevent next day foaming. On the following day, the digestion tubes were placed on a digestion block at 80 °C for one hour and then raised to between 120 °C and 130 °C until a clear solution was obtained [24]. Although nitric acid alone can be used as an oxidizing agent, however, the advantage of this method is due to complete degradation of organic material and safety reasons (reduction of hazards linked to perchloric acid). On the other hand, disadvantages are due to handling of three hazardous acids and higher costs. The digested and transparent solutions were filtered through Whatman (0.45 μm) filter paper into acid-washed volumetric flasks. Double deionized water was used to increase the volume to 50 mL. An Analyst 700 atomic absorption spectrometer (PerkinElmer, Waltham, MA, USA) was used.

### 2.3. Quality Control

For verification purposes and to achieve a high accuracy and precision, a reagent blank sample as well as standard reference soil and vegetable samples (NIST-SRM, 1570a for spinach leaves and SRM 2709 for soil) were included in the digestion procedure [24]. Plant and soil were first analyzed for selected heavy metals according to standard optimum conditions of each metal (Table 1). All chemicals and reagents used in the experiments were of analytical grade and purchased from Merck (Darmstadt, Germany). All glassware used for digestion and preservation of the digested samples were washed with a solution of 10% of nitric acid followed by washing with double deionized water. An average value of triplicates was used to support the interpretation of findings.

**Table 1:** Instrumental analytical conditions associated with the analyses of selected elements.

Element	Acetylene (L/min)	Air (L/min)	Wave Length (nm)	Slit Width (nm)	Lamp Current (Ma)	Detection Limit (µg/L)
Calcium	2.0	17.0	422.7	0.7	10.0	1.5
Cadmium	2.0	17.0	228.8	0.7	4.0	0.8
Iron	2.3	17.0	288.3	0.2	30.0	5.0
Potassium	2.0	17.0	766.5	0.7	12.0	3.0
Magnesium	2.0	17.0	285.2	0.7	6.0	0.2
Manganese	2.0	17.0	279.5	0.2	20.0	1.5
Sodium	2.0	17.0	589.0	0.2	8.0	0.3
Lead	2.0	17.0	283.3	0.7	30.0	5.0
Zinc	2.0	17.0	213.9	0.7	15.0	1.5

### 2.4. Statistical Analysis

All data were statistically analyzed using the statistical software package SPSS 17 (International Business Machines Corporation, Armonk, NY, USA). One-way analysis of variance to confirm the variability and validity of results was performed. The Duncan’s multiple range test was applied to determine significant differences among treatments at a significance level of *p* < 0.05. A linear regression analysis was performed to establish the relationships between heavy metal concentrations in the plant tissue and the corresponding concentrations in the soil.

## 3. Results and Discussion

### 3.1. Effects of Heavy Metals on Plant Biomass

The toxicological effects of Cd, Pb and Zn individually and in combination on the biomass of shoots and roots (fresh and dry weights) of *S. oleracea* were assessed. Results revealed that these heavy metal concentrations have significantly (*p* < 0.05) impacted on the biomass of *S. oleracea*. Shoot and root (fresh and dry) weights were decreased by 25.3, 42.4, 10.1 and 35.1% at the highest dose of Cd exposure as compared to controls treatment, respectively (Table 2). Similarly, the shoot and root (fresh and dry) weights decreased by 25, 47, 8 and 28% at highest dose of Pb exposure as compared to the control treatment, respectively (Table 2). Similar to Cd and Pb, exposure to higher doses of Zn the shoot and root (fresh and dry) weights decreased by 23, 44, 6 and 14% as compared to the controls, respectively (Table 2).

**Table 2:** Reduction (%) in growth parameters of Spinach (*Spinacia oleracea*) due to different heavy metal (cadmium (Cd), Pb (lead) and Zn (zinc)) treatments (in comparison to the controls).

Treatment	Shoot Fresh Weight	Root Fresh Weight	Shoot Dry Weight	Root Dry Weight	Shoot Length	Root Length
Cd 1	7.0 ± 0.10	23.5 ± 0.20	2.5 ± 0.08	21.1 ± 0.19	8.2 ± 0.10	15.9 ± 0.10
Cd 2	18.6 ± 0.11	27.1 ± 0.30	6.3 ± 0.09	28.1 ± 0.20	13.1 ± 0.30	17.7 ± 0.2
Cd 3	25.3 ± 0.09	42.4 ± 0.33	10.1 ± 0.10	35.1 ± 0.30	18.0 ± 0.30	19.7 ± 0.22
Pb 1	5.4 ± 0.12	20.0 ± 0.12	1.90 ± 0.09	9.0 ± 0.17	6.6 ± 0.21	10.8 ± 0.20
Pb 2	18.3 ± 0.20	21.2 ± 0.21	5.9 ± 0.06	14.0 ± 0.25	11.5 ± 0.10	13.3 ± 0.23
Pb 3	24.7 ± 0.31	47.1 ± 0.30	7.6 ± 0.10	28.1 ± 0.40	13.0 ± 0.20	15.8 ± 0.30
Zn 1	3.0 ± 0.01	18.9 ± 0.22	1.8 ± 0.08	8.1 ± 0.20	4.6 ± 0.19	6.0 ± 0.01
Zn 2	17.7 ± 0.13	20.5 ± 0.25	3.8 ± 0.10	10.1 ± 0.21	4.9 ± 0.30	8.8 ± 0.01
Zn 3	23.0 ± 0.38	43.5 ± 0.28	5.7 ± 0.10	14.4 ± 0.12	3.0 ± 0.10	12.7 ± 0.11
Cd 1/Pb 1	10.5 ± 0.19	30.9 ± 0.20	3.5 ± 0.09	28.1 ± 0.30	10.1 ± 0.10	17.1 ± 0.20
Cd 2/Pb 2	27.4 ± 0.20	32.5 ± 0.25	12.1 ± 0.14	35.1 ± 0.32	14.3 ± 0.20	20.9 ± 0.23
Cd 3/Pb 3	30.1 ± 0.29	78.8 ± 0.27	13.9 ± 0.13	42.1 ± 0.23	20.9 ± 0.30	22.0 ± 0.23
Cd 1/Zn 1	6.8 ± 0.12	25.9 ± 0.33	3.0 ± 0.10	25.1 ± 0.11	6.4 ± 0.29	16.1 ± 0.12
Cd 2/Zn 2	19.2 ± 0.22	29.4 ± 0.40	10.6 ± 0.14	32.1 ± 0.12	8.0 ± 0.10	18.6 ± 0.23
Cd 3/Zn 3	28.7 ± 0.38	58.8 ± 0.38	12.2 ± 0.20	43.2 ± 0.30	18.0 ± 0.10	20.0 ± 0.33
Zn 1/Pb 1	6.5 ± 0.12	23.5 ± 0.22	2.5 ± 0.08	15.1 ± 0.15	2.0 ± 0.09	11.5 ± 0.10
Zn 2/Pb 2	20.4 ± 0.23	27.1 ± 0.35	9.7 ± 0.09	17.1 ± 0.23	6.2 ± 0.11	14.6 ± 0.21
Zn 3/Pb 3	26.5 ± 0.30	54.7 ± 0.44	11.4 ± 0.10	32.1 ± 0.33	14.1 ± 0.20	16.2 ± 0.23

The reductions in shoot and root (fresh and dry) weights were 30, 79, 14 and 42%, respectively, if compared to controls, under the influence of the highest dose of Cd mixed with Pb. Similarly, at highest dose of Cd mixed with Zn the shoot and root (fresh and dry) weights decreased by 29, 59, 12 and 43% (Table 2), respectively, as compared to the control. Addition of a high dose of Pb mixed with Zn decreased shoot and root (fresh and dry) weights by 27, 55, 11 and 32%, respectively, if compared to controls (Table 2).

With both Cd and Pb added to soil, the toxicity of spinach biomass was further increased, but was less than the sum of the toxicity for Cd and Pb, when added separately. Toxicity in the biomass of seedlings under the influence of combined Cd and Zn was more than the individual toxicities of Cd and Zn. The combined toxicity of Zn and Pb was less than the toxicity associated with separate Zn and Pb on *S. oleracea* biomass. The dry weights of both shoots and roots were more affected than their fresh seedling weights. The (fresh and dry) root weights were affected more than the (fresh and dry) shoot weights.

### 3.2. Impact of Heavy Metals on Plant Length

Shoot and root lengths of *S. oleracea* were significantly (*p* < 0.05) affected by the addition of individual Cd, Pb and Zn concentrations as well as their mixtures. The increasing concentrations of heavy metals in the soil resulted in a decrease of the shoot and root lengths of *S. oleracea* (Table 2). At highest dose of Cd, the shoot and root lengths decreased by 18 and 20%, respectively, if compared to controls (Table 2). Similarly, at the highest dose of Pb, the shoot and root lengths decreased by 13 and 16%, respectively (Table 2), as compared to controls. At highest dose of Zn, the shoot and root lengths decreased by 3 and 13%, respectively, as compared to controls (Table 2). This reduction in the shoot and root lengths were 21 and 22%, respectively, as compared to control under influence of the highest dose of Cd/Pb. The combined toxicity of Cd and Zn in the shoot and root lengths were 18 and 20%, respectively, if compared to controls. This reduction in shoot and root lengths were 14 and 16%, respectively, under the influence of Pb and Zn combined. Generally, the root lengths were more affected than shoot lengths.

### 3.3. Effects of Heavy Metals on Total Protein, Fiber Characteristic and Moisture Content

**Figure 2:**
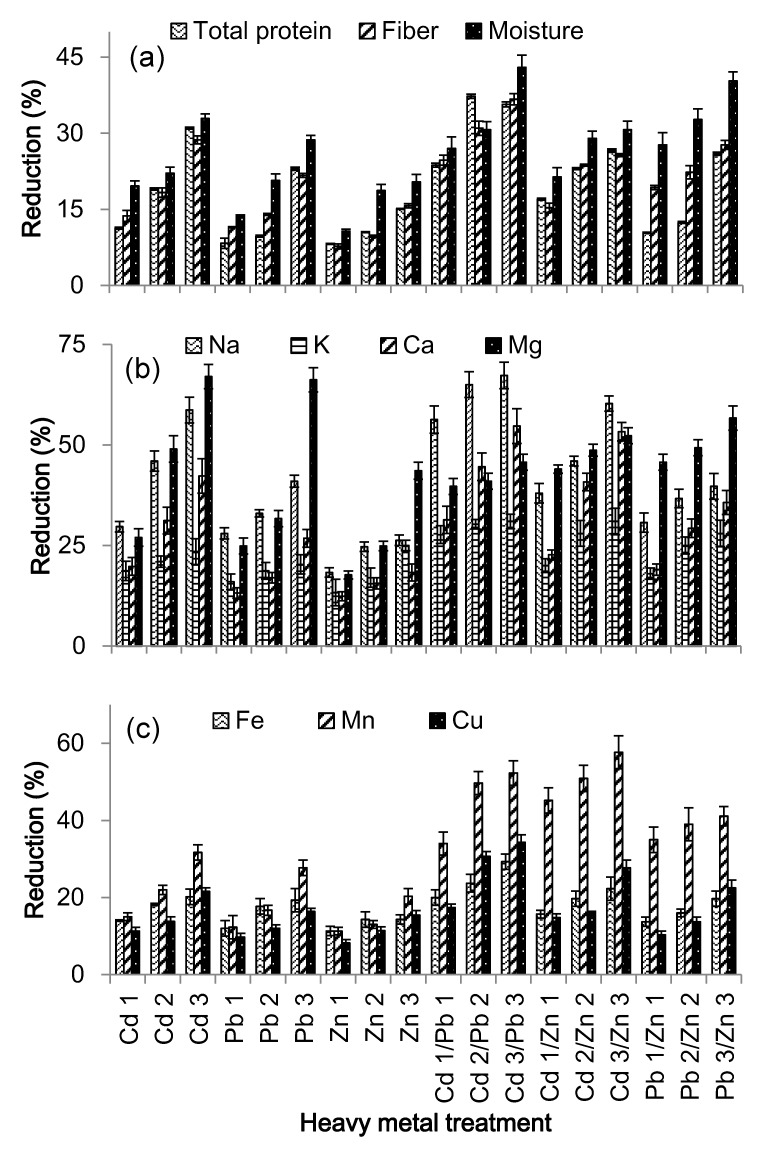
Reduction (%) in (**a**) total protein, total fiber and moisture; (**b**) sodium (Na), potassium (K), calcium (Ca) and magnesium (Mg); and (**c**) of iron (Fe), manganese (Mn) and copper (Cu) determined for Spinach (*Spinacia oleracea*) due to different heavy metal (cadmium (Cd), Pb (lead) and Zn (zinc)) treatments (in comparison to the controls).

Heavy metal concentrations had a significant (*p* < 0.05) adverse impacts on protein, fiber and moisture contents of *S. oleracea*. At highest dose of Cd, the total protein, fiber and moisture contents of *S. oleracea* decreased by 31, 29 and 33%, respectively (Figure 2), if compared to controls. Similarly, at highest dose of Pb, the total protein, fiber and moisture contents decreased by 23, 22 and 29%, respectively (Figure 2), as compared to controls. Addition of a high dose of Zn decreased the total protein, fiber and moisture contents by 16, 16 and 20%, respectively, if compared to controls. This reductions in total protein, fiber and moisture contents were 36, 37 and 43%, respectively, if compared to controls under the influence of the highest dose of combined Cd and Pb. Similarly, at highest dose of Cd and Zn, the total protein, fiber and moisture contents decreased by 27, 26 and 31%, respectively, if compared to controls (Figure 2). Addition of a high dose of combined Pb and Zn decreased the total protein, fiber and moisture contents by 26, 28 and 40%, respectively (Figure 2), as compared to controls. Toxic effects to mixture of Cd and Pb on the total protein, fiber, sodium, potassium, calcium, iron and manganese are more than their individual effects.

In case of moisture content, the combined toxicity of Cd and Pb is less severe than the toxicity due to Pb and more severe than the toxicity associated with Cd. Concerning the calcium and magnesium content of plant, the mixture of Cd and Pb had a less severe impact compared to individual Cd and Pb treatments. The combined toxicity due to Cd and Zn was less severe than the toxicity due to Cd and more than the toxicity of Zn for almost all nutrient components of *S. oleracea*. The toxicity due to Pb combined with Zn was more severe than the toxicity due Pb and Zn alone in case of their influence on the nutrient content.

### 3.4. Effects of Heavy Metals on Nutrient Uptake

The increasing concentrations of heavy metals resulted in the decrease of sodium, potassium, calcium, iron, magnesium, manganese and copper in *S. oleracea* (Table 2). These reductions in concentrations within *S. oleracea* were significant (*p* < 0.05). At the highest dose of Cd, the concentrations of sodium, potassium, calcium, iron, magnesium, manganese and copper reduced to 59, 24, 42, 21, 67, 32 and 22%, respectively (Table 2), as compared to the controls. Similarly, at the highest dose of Pb, the concentrations of these elements showed corresponding reductions of 41, 20, 27, 19, 66, 28 and 16% (Table 2). At the highest dose of Zn, the concentrations of sodium, potassium, calcium, iron, magnesium, manganese and copper showed reductions of 26, 25, 18, 44, 20 and 15%, respectively (Table 2), if compared to the controls. The reductions in concentrations of these elements were 67, 31, 55, 29, 46, 52 and 34%, respectively, if compared to the control under the influence of the highest combined dose of Cd and Pb. The concentrations of sodium, potassium, calcium, iron, magnesium, manganese and copper were 60, 31, 53, 22, 52, 58 and 30%, respectively, if compared to the controls under the influence of the highest combined dose of cadmium and zinc. Similarly, at the highest combined dose of lead and zinc, concentrations of these elements were 40, 28, 36, 20, 57, 41 and 23%, respectively, as compared to the controls.

### 3.5. Up take of Heavy Metals

**Figure 3:**
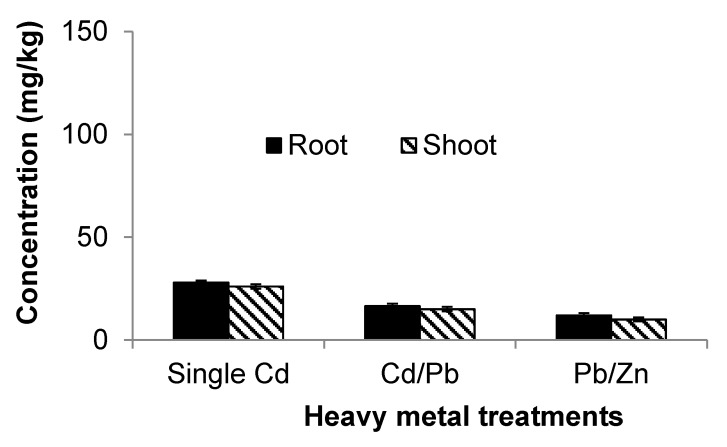
Comparison of cadmium (Cd) uptake subject to different treatments.

Increasing concentrations of Cd in soil (whether as a single contaminant or as part of a mixture with another element) resulted also in a corresponding increase of Cd in the roots (Table 3). Root accumulation of Cd follows the order Cd alone > Cd and Pb combined > Cd and Zn combined (Figure 3). Similarly, it was found that with an increase in concentration of Cd within the roots, its corresponding concentration in the shoots also increased.

The concentrations of Pb within roots showed an increase with increasing Pb concentrations within the soil. The regression analysis showed positive relationships between the concentrations of Pb within the soil and roots for all three treatments; *i.e.*, Pb alone, Pb mixed with Cd and Pb mixed with Zn (Table 3). All correlation relationships for Pb concentrations within roots and shoots are positive. The increasing concentrations of Pb within roots also correspond to increasing concentrations within the shoots.

**Figure 4:**
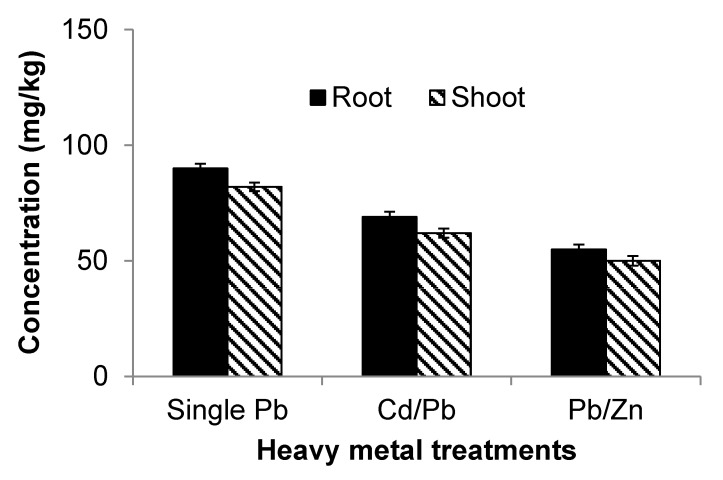
Comparison of lead (Pb) uptake subject to different treatments.

The regression analysis showed strong positive relationships among the Zn concentrations in soil and roots for Zn alone, combined Zn and Cd and combined Zn and Pb treatments (Table 3). Root- accumulated Zn follows this order: Zn alone > Zn combined with Cd > Zn combined with Pb (Figure 4). Table 3 shows relationships between the Zn concentrations within the roots and shoots of *S. oleracea* concerning zinc alone, Cd combined with Zn and Pb combined with Zn treatments. All relationships were positive. An increasing concentration of Zn within roots increases the concentration in the corresponding shoots as well.

Plants grow on metal-contaminated soils simulating soils that have been irrigated with contaminated water for a long time, and accumulate heavy metals in their body tissues [1,2,27,28]. Heavy metals are toxic to plants, subsequently reducing plant yield, affecting leaf and root growths, and inhibiting enzymatic activities [8].

**Table 3:** Linear regressions model for heavy metal concentrations in soils, roots and shoots.

Heavy Metal Uptake	Heavy Metal Treatment	Medium	Coefficient of Determination
Cadmium (Cd)	Cd	Soil to root	0.8037
		Root to shoot	0.9999
	Cd/Pb	Soil to root	0.9671
		Root to shoot	1.0000
	Cd/Zn	Soil to root	0.9976
		Root to shoot	0.9995
Lead (Pb)	Pb	Soil to root	0.9001
		Root to shoot	0.9991
	Pb/Cd	Soil to root	0.8150
		Root to shoot	0.9075
	Pb/Zn	Soil to root	0.9450
		Root to shoot	0.9830
Zinc (Zn)	Zn	Soil to root	0.7076
		Root to shoot	1.0000
	Zn/Cd	Soil to root	0.8283
		Root to shoot	0.9995
	Zn/Pb	Soil to root	0.9340
		Root to shoot	0.9994

Cadmium inhibits plant growth, and its toxicity increases with increasing Cd concentration in soil. In the present study, increasing concentrations of Cd significantly (*p* < 0.05) reduced shoot and root fresh and dry weights. The results of this study support previous research [25,33,34,35,36,37,38]. However, the results of the present study are not consistent with other published findings [39,40]. Increasing concentrations of Cd in soil led to toxicity in plant biomass and plant lengths of *S. oleracea*, which is in agreement with previous work [25,37]. Cadmium toxicity was more severe within roots in terms of both biomass and length. Roots are more sensitive than shoots, because they are part of plants, which come into contact with toxic substances first. Researchers [11] reported that a reduction in the formation of new cells under the influence of Pb and Cd leads to a reduction in shoot and root lengths.

Spinach was suffering from toxicity with increasing concentrations of Pb in terms of plant biomass and length. Increasing concentrations of Pb significantly (*p* < 0.05) reduced plant biomass and length. Other researchers [25,35,36] observed decreases in plant shoot and root growths with increasing concentrations of Pb in the growth medium.

Zinc is an essential element for plants, but its excess can significantly damage plants [4]. This study indicated that increasing concentrations of Zn are responsible for increased toxicity in *S. oleracea*. Shoot and root (fresh and dry) weights reduced with increasing concentrations of Zn. Zinc reduced plant biomass, because it led to a deficiency of macro-nutrients such as phosphorus [34]. Researchers [36] also found reductions in growth of corn with increasing concentrations of Zn.

**Figure 5:**
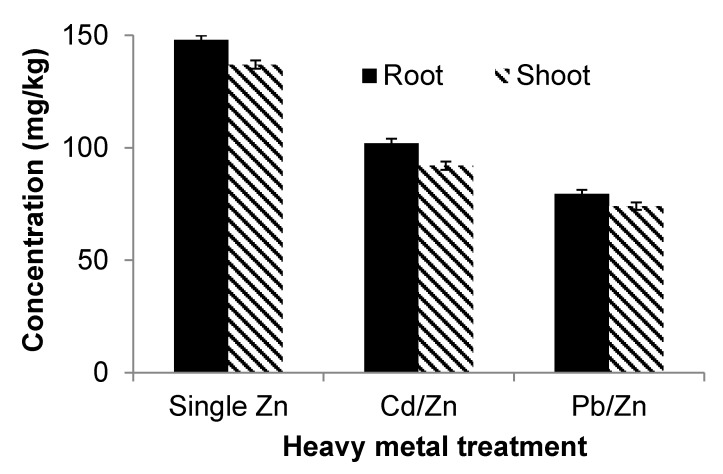
Comparison of zinc (Zn) uptake subject to different treatments.

The combined toxicity of Cd and Pb in terms of biomass and length was found to be more severe than the toxicity of Cd and Pb alone, but was less than the additive toxicity of the two heavy metals alone. The uptake results showed that in combination, Cd decreased the uptake of Pb (Figure 5) and Pb lowered the uptake of Cd (Figure 4). Although both Cd and Pb are toxic on their own, they decrease the uptake of each other if combined. It follows that the combined toxicity does not equal the additive of both toxicities. Some studies [37] have reported similar results for broccoli at low Cd concentrations, while others [33] produced the same result in studying Cucumber. The combined toxicity of Cd and Zn is more severe than the individual toxicity of Cd and Zn, but less than the additive toxicity of the two heavy metals alone. The combined toxicity is not the sum of both toxicities, because Zn and Cd both reduce the uptake of each other by plants [38].

The combined toxicity of Cd and Zn is more than the individual toxicities of Zn and Cd in terms of biomass plant length. The result is consistent with previous findings [39]**.** The combined toxicity of Pb and Zn in terms of total biomass and length is more than the corresponding individual toxicities, but less than the sum of the individual toxicities of Zn and Pb. The reason for the reduction in toxicological effect of combined Pb and Zn is the antagonistic effects of Zn and Pb. Lead decreases the uptake of Zn (Figure 5) and Zn reduces the uptake of Pb (Figure 4).

As the concentration of Cd increases, Pb and Zn decrease the total protein contents in plants. The results of this study show a drop in the total protein content in plant shoots with increasing concentrations of Cd, Pb and Zn alone and also with respect to their mixtures; *i.e.* combinations of Cd and Pb, Cd and Zn and Pb and Zn. The results are consistent with those of previous studies [4,40,41]. Other researchers [40,42] found a reduction in protein content for *Daucus carota* (carrot) and *Helianthus annuus* (sunflower) with increasing concentration of Cd in the growth medium. A high dose (1500 μM) of Pb was responsible for 77% reduction in protein content in *Brassica juncea* (mustard greens) [43]*.*

Previous studies reported a reduction in protein content in algae and *Brassica napus* (rapeseed) as Zn increased [4,44,45]. There are many reasons for the drop in protein content with heavy metals. A drop may be due to the accelerating degradation of protein with increasing protease activity [46] or disturbance of nitrogen metabolism in the presence of heavy metals such as Cd and Pb. The protease activity increases in stress conditions [43] like the presence of heavy metals in the growth medium. According to previous work [47], heavy metals such as Cd and Pb disturb nitrogen metabolism, which further decreases the synthesis of protein. Heavy metals including Cd are responsible for the reduction in photosynthesis, which reduces the synthesis of protein [40].

The results of the present study showed that an increase in the concentrations of heavy metals resulted in a decrease of sodium, potassium, calcium, iron, magnesium, manganese and copper in *S. oleracea*. The results are consistent with previous findings. An excess of Zn decreased the uptake of elements like magnesium, manganese, copper and iron in plants [4]. An increasing concentration of Cd interfered with other elements like potassium, calcium and magnesium by disturbing their distribution in plant parts and also decreasing their content in plant tissue [40].

Scientists [48] found a decrease in manganese concentration in barley plants with increasing concentrations of Cd. Cadmium decreased the uptake of iron [49], potassium, manganese and calcium [44]. The toxicity in terms of reduction uptake rates of essential elements by Cd is more severe than the individual toxicity due to Pb and Zn. This might be attributed to a higher toxicity and rate of accumulation of Cd in plants, especially in roots.

Others [45] found bioaccumulation coefficients for Cd of up to 1100 in shoots and 6700 in roots at a concentration of 0.1 µg Cd/mL in soil. The reduction in the uptake of elements was greater for a combination of Cd and Pb treatments in comparison to their individual treatments, but it was less than the sum of both toxicities. This might be due to a reduction in the uptake of both heavy metals. Similarly, the combined toxicity in terms of reduction of metals uptake for combined Cd and Zn treatment was less severe than due to Cd alone, but more than for Zn alone. Zinc reduced Cd uptake [40], so that the corresponding toxicity is also reduced. Similarly, the combined toxicity due to Pb and Zn was less than the individual one for Pb.

High concentrations of Cd, Pb and Zn were found in roots. These results are in agreement with previous reports [50] indicating the presence of high concentrations of Cd in *Solanum lycopersicum* (tomato) roots. Similarly, others [32] observed high concentrations of Pb in roots of plants. Roots showed high concentrations of heavy metals if compared to other plant parts, because heavy metals come into contact with the roots of plants first [51].

## 4. Conclusions and Recommendations

Plant exposure to heavy metal contamination resulted in severe toxicity of *S. oleracea*. Results revealed that Cd and Pb treatments even at low concentrations and Zn at high concentration induces a significant (*p* < 0.05) reduction in all growth parameters (shoot and root lengths, biomass and number of leaves) as well as total protein content, fiber, moisture content and minerals (Na, K, Ca, Fe, Mg, Mn and Cu) of *S. oleracea.* The impacts of all selected heavy metals significantly depended on their concentrations in the plant tissues. The results of the combined toxicity showed antagonistic affects. The uptake rate of Cd by *S. oleracea* was higher compared to previous studies. This was reflected in the growth of the plants. Further work in the field using real and not simulated wastewater contaminated by heavy metals is recommended. A greater variety of crops grown in different geographical regions would be helpful. However, such studies are likely to result in highly variable data and may take years or even decades to conclude.
